# First Report of Acute Myocarditis Post-Pfizer-BioNTech COVID-19 Vaccination in the Kingdom of Bahrain

**DOI:** 10.7759/cureus.20313

**Published:** 2021-12-09

**Authors:** Tarique S Chachar, Nooraldaem Yousif, Leena Sulaibikh, Fuad Abdulqader, Manaf Alqahtani

**Affiliations:** 1 Cardiology, Mohammed Bin Khalifa Specialist Cardiac Centre, Riffa, BHR; 2 Interventional Cardiology, Mohammed Bin Khalifa Specialist Cardiac Centre, Riffa, BHR; 3 Non-Invasive Cardiology, Mohammed Bin Khalifa Specialist Cardiac Centre, Riffa, BHR; 4 Infectious Disease, Bahrain Defence Force Royal Medical Services, Riffa, BHR

**Keywords:** sers cov 2, myocarditis, vaccination, corona virus, covid-19

## Abstract

We present a case of a 24-year-old male patient who presented to our institution five days after receiving his first dose of Pfizer-BioNTech vaccine to rule out acute coronary syndrome due to chest pain along with troponin increase and ECG changes. Acute coronavirus disease 2019 (COVID-19) infection was excluded based on a negative real-time reverse transcription-polymerase chain reaction (RT-PCR) test of specimens acquired using nasopharyngeal swabs for severe acute respiratory syndrome coronavirus 2 (SARS-CoV-2), and all other viral serologies were found to be negative. Coronary angiogram showed normal coronaries, and the presence of late gadolinium enhancement, which is indicative of myocarditis, was identified using cardiac magnetic resonance imaging (MRI). Our case report raises concern that the COVID-19 vaccine may cause myocarditis as a rare side effect.

## Introduction

The novel coronavirus severe acute respiratory syndrome coronavirus 2 (SARS-CoV-2), also known as coronavirus disease 2019 (COVID-19), originated in December 2019 in Wuhan, China. It has ever since rapidly spread worldwide, causing morbidity and mortality in its way. In March 2020, the World Health Organization (WHO) declared the COVID-19 outbreak a pandemic [1,2]. In order to control the spread of "COVID-19", there was a need to develop vaccines very quickly and it culminated in the discovery of several potential vaccine candidates. Regulatory authorities throughout the world then fast-tracked approvals for these vaccines under the emergency use of authorization in December 2020 [1]. Furthermore, companies have been racing to find a vaccine for the coronavirus, which has killed over 1.5 million people and infected more than 65 million since it emerged in China in December of last year [3].

Bahrain approved emergency use authorization of the Pfizer-BioNTech coronavirus vaccine, becoming the second country after Britain to green-light the vaccine [3,4]. The approval of the Pfizer/BioNTech vaccine added a further important layer to the kingdom's national COVID-19 response.

Vaccines, like any medication, can have side effects. Serious side effects to vaccines, if they occur, are rare. In this paper, we report the first case of acute myocarditis after the administration of Pfizer-BioNTech vaccine in the Kingdom of Bahrain.

## Case presentation

**Figure 1:**
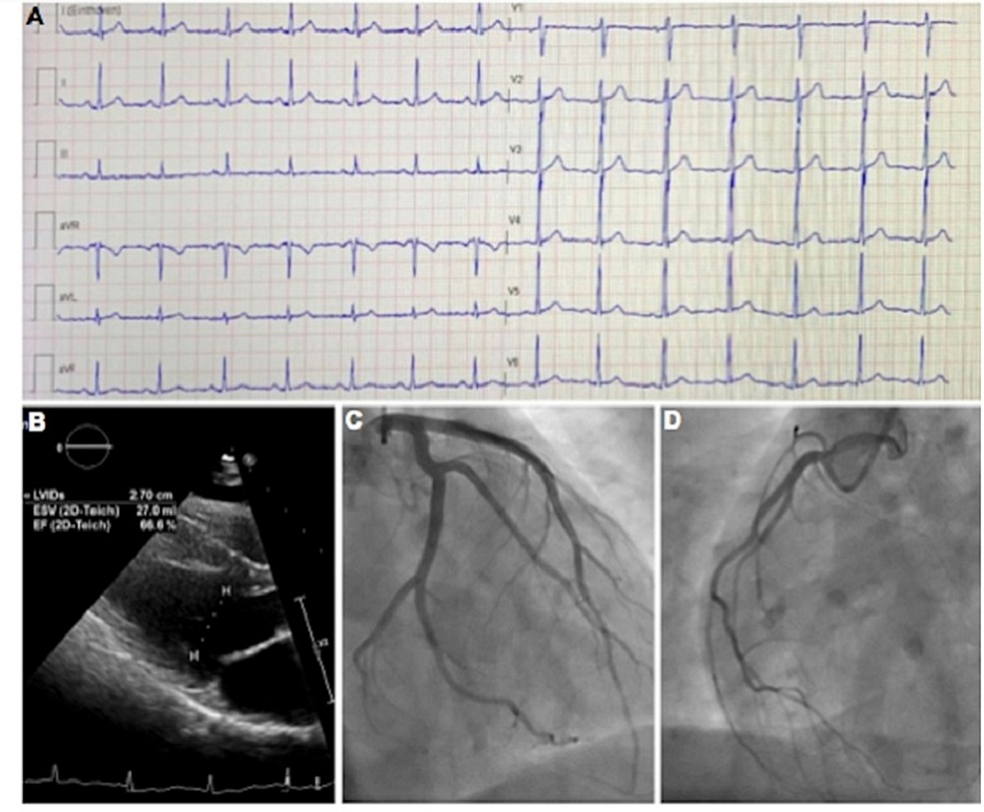
A: 12-lead electrocardiogram showed subtle ST elevation in the precordial leads. B: Transthoracic echocardiogram parasternal long-axis view showed normal left ventricular systolic function. C, D: Coronary angiogram showed normal coronaries.

A previously healthy 24-year-old Saudi male patient developed chest pain five days after receiving the first dose of the Pfizer-BioNTech (BNT162b2) vaccine. There was no history of viral infection, recent or distant, and no known exposure to confirmed COVID-19 patients. He was transferred to our cardiac center to rule out acute coronary syndrome (ACS). Initial electrocardiogram (ECG) showed subtle ST elevation in the precordial leads (Figure 1A). His physical examination was deemed unremarkable. Laboratory findings showed considerably high levels of Troponin-I (2.1 ng/ml; normal range 0.01-0.03 ng/ml), creatinine kinase (568 IU/L; normal range 20-200 IU/L), and C-reactive protein (7 mg/dl; normal range 0-5 mg/L). The complete blood count, renal function, electrolytes levels, as well as liver function tests, iron studies, and serum electrophoresis, and immunofixation were all within normal limits. Furthermore, a nasopharyngeal swab was negative for severe acute respiratory syndrome coronavirus 2 (SARS-CoV-2). All other viral tests were negative, including parvovirus B19, Epstein-Barr virus, cytomegalovirus, adenovirus, influenza, herpes simplex 1 and 2, coxsackievirus, hepatitis, human immunodeficiency virus, and Lyme antibodies. Transthoracic echocardiography (TTE) revealed a preserved left ventricular ejection fraction (LVEF 55%) and no regional wall motion abnormalities (Figure 1B). Coronary angiogram revealed normal epicardial coronaries (Figure 1C and Figure 1D).

**Figure 2:**
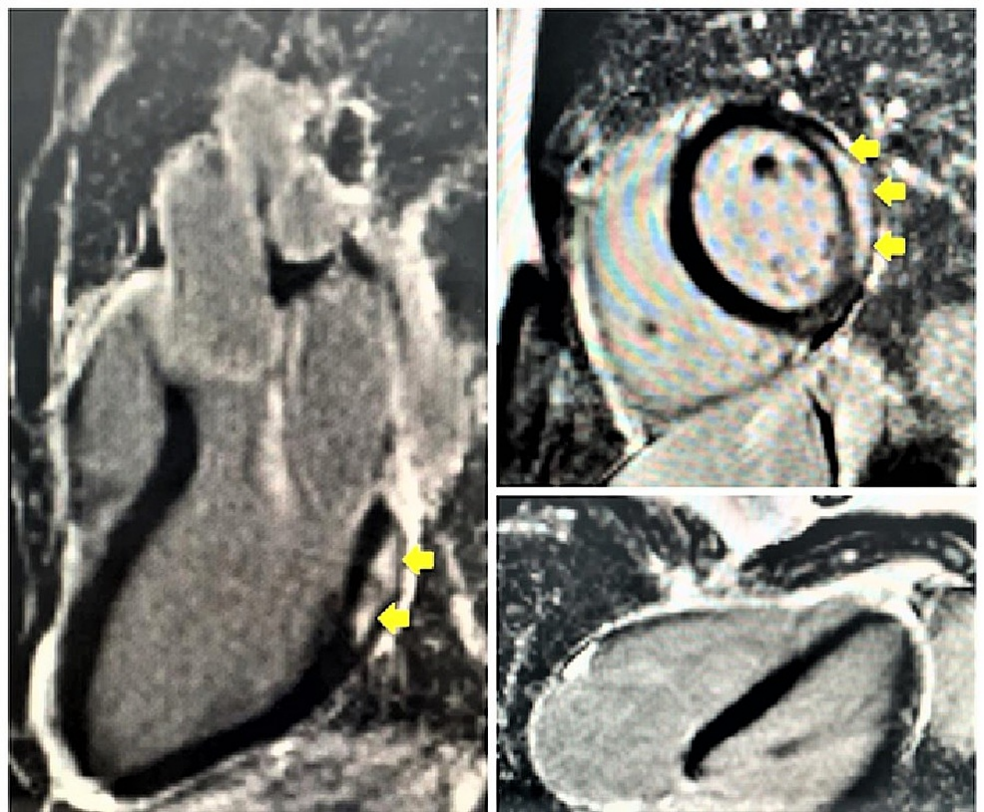
Cardiac magnetic resonance (CMR) imaging showed sub-epicardial gadolinium enhancement (yellow arrows) in the basal to mid-lateral and inferolateral walls (non-ischemic pattern) suggestive of myocarditis.

Cardiac MRI demonstrated subepicardial gadolinium enhancement in the basal to mid-lateral and inferolateral walls (non-ischemic pattern) suggestive of myocarditis. The patient was discharged two days after being diagnosed with myocarditis on perindopril 2.5 mg OD and bisoprolol 2.5 mg OD. He reported being asymptomatic, good functional class, and able to exercise normally after a four-week follow-up through phone consultation owing to hospital COVID-19 protocols (Figure 2).

## Discussion

Clinical trials conducted by Pfizer and BioNTech demonstrated that mRNA vaccines increased systemic reactogenicity and immunogenicity in younger study participants. According to one study, 41.5% of teenagers reported chills after receiving the vaccine, compared to 35.1% of patients aged 18-55 years. One month following the second dosage, children 12-15 years of age showed a greater geometric mean titer (GMT = 1,239.5) compared to individuals 16-25 years of age (GMT = 705.1) in terms of immunogenicity [4].

Common side effects include pain, redness, and swelling at the injection site, along with other mild symptoms, such as tiredness, headaches, chills, muscle aches, and fever. The following symptoms could indicate that the body is becoming more immune to the infection to protect itself [5].

Since April 2021, Pfizer-BioNTech reported rare but more serious adverse events, such as myocarditis and pericarditis, to the Vaccine Adverse Event Reporting System following mRNA COVID-19 vaccination. According to the Israeli Ministry of Health, 62 people who received the COVID-19 vaccine developed myocarditis [6]. There were only six cases reported after the first dosage of mRNA vaccinations, while most cases were diagnosed after the second dose. When the prevalence rate was compared, it is seen that the cases of myocarditis in the general population was 1 in every 100,000 individuals, but in individuals between 16 and 30 the myocarditis prevalence rate was 1 in every 20,000 individuals indicating the fact that younger individuals are more prone to myocarditis following the administration of COVID-19 mRNA vaccines [5,6]. 

According to the US Department of Defense, 14 people got myocarditis after getting COVID-19 mRNA vaccinations, 13 of them after the second dose. At 0.52/100,000, there was an incidence rate of three of the 2.7 million immunized military members who received the Pfizer/BioNTech vaccination and eleven who received the Moderna vaccine [7].

Pfizer-COVID-19 BioNTech's vaccine is the only vaccine currently approved by Bahrain's national medical task force for combating the coronavirus for adolescents 12-17 years of age [8]. COVID-19 vaccinations and myocarditis may have a temporal link, and proper diagnosis might protect healthy teenagers and young adults with chest pain and ECG ST elevation from unnecessary invasive medical procedures like coronary angiography.

## Conclusions

To the best of our knowledge, this is the first reported case of acute myocarditis post-Pfizer-BioNTech COVID-19 vaccination in the Kingdom of Bahrain. The mechanisms underlying COVID-19 vaccines-associated myocarditis are yet to be understood. Though rare, physicians and healthcare providers are urged to foresee the possibility of myocarditis in adolescents and young adults who experience chest pain, troponinemia, and ST elevation following COVID-19 vaccination and consider non-invasive diagnostic approaches to rule out ACS.
